# Experiments to Prove That the Capillaries of the Lungs Do Not Anastomose

**Published:** 1868-03

**Authors:** G. P. Cammann


### Experiments to prove that the Capillaries of the Lungs do not Anastomose.

BY G. P. CAMMANN, M. D.

As it is still a question among anatomists,* whether the Pulmonary Capillaries do or do not Anastomose, the following experiments may not be without their value:

*Experiment* 1.—We injected water colored with gamboge into a branch of the pulmonary artery going to a section of lung, and found that a defined portion became moderately distended with the fluid; the water then commenced flowing from the accompanying *bronchus*† *(?)* and vein. The latter being secured by ligature, we continued to force in the injection, until this portion of lung became very tense. The injection continued confined to the same original extent of lung; the surrounding parts remained perfectly flaccid, and not a drop of the fluid escaped from any of the *bronchi* or veins.

*Exper.* 2.—A similar injection was forced into a branch of the *pulmonarg vein.* The fluid flowed only from the accompanying bronchus and artery. (The injection entered with such facility as to show that its course was not interrupted by any valves.)

* Reisseissen, Marshall Hall, and others.

† How can the injection into a pulmonary artery or vein escape "from the accompanying bronchus" unless by rupture of the bronchial mucus or nosicular capillaries? Is this what the writer means? H. N. EASTMAN.

*Exper.* 3.—We moderately inflated a section of lung through a bronchial tube and secured it by ligature. The accompanying pulmonary artery was then injected, and it was found that the injection extended exactly as far as the inflated portion of lung, following closely the indentations so marked at the boundaries of the bronchial ramifications; the surrounding parts remaining flaccid.

*Exper.* 4.—We substituted the vein for the artery with similar results.

Having, to our entire satisfaction, repeated the above experiments a number of times, we proceeded from the larger arteries and veins to the more minute; gradually approaching the periphery or free border of the lung, and at each advance necessarily including a smaller section. We found in these experiments, as in the former, that the injection of colored water remained confined to the ramifications of the artery or vein under examination, and did not interfere with the neighboring branches. We thus pursued the arteries and veins until they became so minute that it was impossible to insert the finest tube. In other words, we approached so near the terminal branches as to inject only a lobule or a part of a lobule.

To confirm the preceding experiments, and place the matter beyond doubt, we had recourse to another series.

*Exper.* 5.—We injected a moderately-sized pulmonary artery with a strong solution of tartaric acid, and continued to fill the vessel, until from the accompanying bronchus and vein there streamed out a fluid which, when tested with bicarbonate of soda, proved to be the acid.

*Exper.* 6.—Having previously secured by ligature the accompanying bronchus and vein, we injected a branch of the pulmonary artery with the acid solution, until the portion of lung to which it was distributed became distended and tense. We then, in a similar manner, injected an adjoining branch of the artery with a strong solution of the bicarbonate of soda, and applied force almost sufficient to rupture the tissue. The two solutions were apparently in close contact, yet not the least chemical action ensued, although we kept the vessels thus distended until we were apprehensive that the chemicals acting through the tissues might lead into error. Neither in this nor in the former experiment did any liquid come from the neighboring vessels.

*Exper.* 7.—When the two solutions were injected successively into the artery and vein accompanying the same bronchus, the lung was rent in every direction by the disengaged carbonic acid.

*Exper.* 8.—We pursued our investigations from the larger branches of the pulmonary vessels to the more minute, and carried them down as far as practicable.

If anastomosis of the capillaries had existed, the effervescence from the soda and acid would have burst the pulmonary tissues as in experiment 7.

As the preparations from the above experiments were of a perishable nature, we injected sections of lung with mucilage of gum Arabic, colored with indigo, and then suspended them in alcohol. The injection passed only to the lobule or lobules to which the vessel was distributed. We possess many of these preparations, in which portions of lung have been injected, the intermediate spaces being entirely free. The surrounding lung remained perfectly flaccid and free from discoloration, even when a degree of force was employed sufficient to rupture the capillaries and form collections of injections either in the substance of the tissue or beneath the pleura. A distinguished pathologist observed this, on submitting sections of the lung to a powerful microscope. So perfectly distinct were the lines of demarcation between the injected and uninjected portion of lung, that we could readily dissect the one from the other, without causing the least escape of fluid. These preparations strikingly illustrate the perfect fidelity with which the experiments had been performed.

In performing these experiments, certain precautions were necessary to ensure success. 1. While cutting off the piece of lung for experiment, care was taken not to draw down the edges, lest the vessels to be injected should be on the apex of a cone, as the terminations of some of the lateral vessels might thus be above the incision. 2. The piece of lung was so held that the colored water flowing from the accompanying bronchus and vein could not run into the neighboring vessels. 3. The surface of the cut lung was wiped so dry that any oozing of fluid could be detected. 4. The injection was passed in very slowly, in order that none of the capillaries or tissues might be ruptured. The liquid was entered rather faster than it flowed from the accompanying vessels. 5. The bronchus and vein were not closed too soon, lest the air, which was, in some cases, forced before the injection, might rupture the capillaries. 6. On approaching as near as possible the periphery of the lung, and after inserting the tube, by compressing with spring-forceps some of the branches of the vessels under examination, a very small portion of a lobule could be injected. 7. In the small lobes of the lungs of the sheep the vein runs superficially for an inch or two, it afforded great satisfaction to observe the colored injection as it returned by the vein, and which continued to flow for some time before it escaped from the accompanying bronchus, proving that it must have passed through the capillaries; and if there had been any communication with the neighboring capillaries, they would have become distended. 8. The warmest weather of summer was found to be the best season for making experiments and preparations. At this time of the year the lungs retain their elasticity, whereas they become dry and do not freely accommodate themselves to the injection after being hardened by a reduction of temperature and then subjected to artificial heat.

We have thus ascertained that there is no anastomosis of the capillaries of one branch of an artery or vein, however minute, with the capillaries of any other branch in the pulmonary circulation; or in other words, that the capillaries of each minute branch of an artery or vein are perfectly isolated and independent of any other capillaries for a collateral supply of blood. We hold it, then, as proved, that there is no anastomosis between the capillaries of one lobule with that of another, nor, as far as analogy will warrant, between the capillaries of one minute or terminal artery with those of another. If any additional confirmation were required, it might be furnished by an examination of the preparations, when it could be seen that the interstitial cellular membrane, even of the most minute lobules, remain perfectly free from any discoloration from the injection.

If we allow the structure of the pulmonary blood-vessels to be as it is generally supposed, and the anastomosis of their capillaries to be as intimate as Dr. Marshall Hall and others describe, we would expect that almost every case of profuse hæmoptisis from pulmonary apoplexy, congestion, or any other cause, most necessarily prove fatal,* from the continual action of the lungs pump-

* We would certainly anticipate such a result, when it is remembered with what facility the injections entered the bronchi.

ing, as it were, the blood from the vessels so easily supplied by the very numerous and freely-communicating capillaries, and from the difficulties which would thus be presented to the formation of coagula. Now, we know, on the contrary, that immediate danger is comparatively slight, notwithstanding, as stated by Bichat, and verified by others, that the lungs, especially when inflamed, are more frequently than any other organs, flooded with an immense quantity of blood. Bichat well says, "Voyez le poumen d'un péripneumonique; en le fendant vous diriez au premier coup d'œil, que ce sont les solides qui y sont augmentés, il a souvent comme l'aspect du foie dans la masse pesante qu'il représente; mais mettez-le macérer, blentôt, tout s'echappera en fluides. Or, examinez comparativement la peau, l'estomac, le foie, les reins, etc., devenus le siége d'unee inflammation aiguë qui a fait succomber le sujet; ils ne présentent rien d'approchant de ce surcroit énorme de fluid dont le poumon inflammé dans sa substance est surchargé." Again; what prevents the occurrence of exhausting hemorrhage when there are abscesses and excavations in the lungs, surrounded by perfectly healthy structure, without even the intervention of false membrane? According to Baillie, this is "principally the case when the abscesses are small and placed at a considerable distance from each other." Yet, even then, we would suppose that the blood would be continually forced out into the cavity by the action of the lungs, while the free communication of the capillaries would furnish an ample supply. The preceding remarks are also applicable to an excavation from pulmonary gangrene; for, as Andral says, "the parietes of this cavity are, in general, not lined by any false membrane; the pulmonary parenchyma which surrounds it is in some cases perfectly healthy."

In the pathological conditions of the lungs there are appearances which are very peculiar, viz: the abrupt and perfectly-defined margin that frequently exists between the healthy and diseased parts. These singular boundaries, as if marked out with a pencil, are found in no other organ, except, perhaps, in the brain. In inflammation, or as the effects of inflammation, in other organs, the surroundiug tissue is involved; and as we recede from the point of most active disease, the remote parts are less affected, but there are no lines of demarcation; indeed, we can scarcely pronounce where a healthy condition commences, whereas, in very many of the diseases of the lungs, the separation between the morbid and healthy parts is perfectly defined, as has been remarked by every pathologist. Addison says that, "especially in certain atonic forms of pneumonia, these changes are confined to individual lobules, more or less remote from each other, the common cellular membrane forms a distinct boundary to the inflammation." Grisolle speaks of isolated lobules being the seat of pneumonia; and Andral alludes continually to this disease as being circumscribed. With respect to pulmonary apoplexy, Laennec observes, that "it is always very exactly circumscribed, the induration being as considerable at the point of termination as in the centre. The pulmonary tissue around is quite sound and crepitous." Andral makes the same remark. He also, in his Clin. Méd., has very interesting observations and facts upon the lobular nature of tubercles, but they are too long to be transcribed.

The experiments of Dr. Marshall Hall on the batrachia, will not apply to warm-blooded animals. In Roget's Animal and Vegetable Physiology, it is beautifully shown how, as we ascend from the lower order in the scale of beings to the most perfectly organized, the pulmonary structure materially varies, so that analogy will not hold good between the different orders of beings. He observes, that in the frog a limited portion only of the blood thrown out from the heart goes to the lungs, so that any inconvenience from intimate communication of the capillaries is prevented; and proceeds to show the perfect and well-developed structure of the lungs of the mammalia. He states, also, that the torpid and cold-blooded reptiles are separated from the mammalia by a very wide interval; for though the former respire air, that air influences but a part of the blood, as the pulmonary is only a branch of the general circulation.

It is allowed by the best anatomists and physiologists, that the capillaries are differently arranged in each organ, being so modified as to accommodate themselves to the tissues through which they ramify. Bichat considers it certain that their distribution and formation differ in the tendons, aponeuroses, muscles, etc.

If we admit that there is no anastomosis between the capillaries of the lungs, we can satisfactorily explain many points in the physiology and pathology of the pulmonary organs. 1. We can easily perceive how, in pulmonary hemorrhage, the arterioles or capillaries, having no collateral supply of blood to instantly rush in and keep them distended, readily contract, and allow the formation of coagula, which effectively prevent excessive loss of blood. 2. We can explain why, in small abscesses where there is not a false membrane between the parietes and healthy structure, there is not a continual flow of blood into the cavity, for as the capillaries discharge themselves they shrink; although in large cavities where, from extent of surface, an exhausting hemorrhage might occur, nature provides a false membrane. 3. We can read understandingly the facts recorded by Bayle, Laennec, Andral, Barth, and indeed by all the correct and skillful pathologists. We understand why pulmonary inflammation, congestion, apoplexy, gangene, etc., are so exactly circumscribed and defined; and why in inflammation we do not, as in other tissues, always observe a gradation in the degree of engorgement, as we recede from the centre of disease. We find the correctness of Andral, when he describes inflammation as attacking isolated points of the pulmonary tissue; and when he speaks of even vesicular pneumonia, of which Grisolle observes, that "Billiet and Barthez seem to show that this lesion, described by Andral, is only vesicular bronchitis, in which a portion only of the pulmonary vesicles are iuflamed and distended with puriform fluid." We see reasons for Andral's statement, that "even in those parts where the hepatization seems most perfect, it rarely happens that some small bronchial tube may not be found still permeable to air; and we sometimes find, that when the lobe of a lung which appeared uniformly hepatized throughout, is dried and carefully examined, we can discover some capillary tubes and air-cells which, instead of having their calibre diminished, are very considerably dilated, and are at the same time free from any appearance of congestion." 4. We can understand when we turn to physical signs, why the crepitous râle is so frequently accompanied by the respiratory murmur. We also ascertain why crepitous râle without respiratory murmur, shows very considerable engorgement; and when respiratory murmur is present, much of the lung is still healthy. We likewise advance towards an explanation of lobular pneumonia in children, and account for the frequent relapse and only apparent convalescence in pulmonary inflammation, from small lobules and vesicles remaining in a condition of active disease, after all physical and rational signs of functional derangement have dissappeared.—Finally, we comprehend the cause of the following phenomena observed by Stokes: "I have frequently seen," says he, "all the signs of solidification subside within two days, and have even observed great modifications in the course of a few hours. * * * On this subject more extensive observation is wanting."

We have thus demonstrated how, by being composed of an aggregate of isolated portions, the lungs are protected from the extension of disease; and how, but for this safeguard of nature, organs so essential to existence would be more liable to permanent injury, when a portion of their tissue is incapable of performing its functions.

